# Discontinuation of imatinib in patients with oligometastatic gastrointestinal stromal tumour who are in complete radiological remission: a prospective multicentre phase II study

**DOI:** 10.2340/1651-226X.2024.39851

**Published:** 2024-05-07

**Authors:** Ivar Hompland, Kjetil Boye, Anne Marit Wiedswang, Andri Papakonstantinou, Bård Røsok, Heikki Joensuu, Øyvind Bruland

**Affiliations:** a Department of Oncology, Oslo University Hospital, Oslo, Norway; b Department of Radiology, Oslo University Hospital, Oslo, Norway; c Department of Breast Cancer, Endocrine Tumors and Sarcoma, Karolinska University Hospital, Stockholm, Sweden; d Department of Oncology-Pathology, Karolinska Institute, Stockholm, Sweden; e Department of Hepato-Pancreatic-Biliary Surgery, Oslo University Hospital, Oslo, Norway; f Comprehensive Cancer Center, Helsinki University Hospital and University of Helsinki, Helsinki, Finland; g Institute of Clinical Medicine, University of Oslo, Oslo, Norway

**Keywords:** Gastrointestinal stromal tumour, GIST, Imatinib, oligometastatic disease, survival, quality of life

## Abstract

**Introduction:**

Metastatic gastrointestinal stromal tumour (GIST) is considered incurable, and life-long treatment with tyrosine kinase inhibitors is recommended. We investigated whether selected patients with metastatic GIST may remain in durable remission despite imatinib discontinuation.

**Patients:**

In this 1-group, prospective, multicentre phase II trial selected patients with oligometastatic (≤3 metastases) GIST discontinued imatinib treatment. Eligible patients had been treated with imatinib >5 years without progression and had no radiologically detectable metastases after metastasectomy, radiofrequency ablation (RFA) or complete response to imatinib. The primary endpoint was progression-free survival (PFS) 3-years after stopping imatinib. Overall survival (OS) and quality of life (QoL) were secondary endpoints.

**Results:**

The trial closed prematurely due to slow accrual. Between January 5, 2017, and June 5, 2019, 13 patients were enrolled, of whom 12 discontinued imatinib. The median follow-up time was 55 months (range, 36 to 69) after study entry. Five (42%) of the 12 eligible patients remained progression free, and seven (58%) progressed with a median time to progression 10 months. Median PFS was 23 months and the estimated 3-year PFS 41%. Six of the seven patients who progressed restarted imatinib, and all six responded. Three-year OS was 100%, and all patients were alive at the time of the study analysis. QoL measured 5 and 11 months after discontinuation of imatinib demonstrated improvement compared to the baseline.

**Interpretation:**

A substantial proportion of selected patients with oligometastatic GIST treated with imatinib and metastasis surgery/RFA may remain disease-free for ≥3 years with improved QoL after stopping of imatinib.

## Introduction

Imatinib has markedly improved overall survival (OS) of patients with advanced gastrointestinal stromal tumour (GIST) with median OS of ≥5 years since starting of imatinib [1, 2]. In oligometastatic GIST, defined as ≤3 detectable metastases, even about 70% 10-year OS can be expected [2]. Though long survival times are achievable, metastatic GIST is considered incurable, and GIST patients are recommended to continue imatinib and other tyrosine kinase inhibitors indefinitely in the absence of disease progression [3]. This recommendation is largely based on the results of the BFR14 trial, where patients with metastatic GIST who were responding to first-line imatinib were randomised to continue or to stop imatinib [4–6]. Nearly all who stopped progressed within 2 years regardless of whether they had been on imatinib 1, 3 or 5 years prior to imatinib discontinuation. Importantly, discontinuation did not negatively influence OS, probably because almost all patients who resumed imatinib had a second response to imatinib.

Approximately 30% of patients with oligometastatic lung metastases from sarcoma who undergo metastasectomy become long-term survivors [7]. Patients with metastatic GIST who responded to imatinib and underwent metastasectomy had favourable survival outcomes in retrospective studies [8–11] and in two prospective trials [12, 13]. Conceptually, surgical removal of metastases may reduce the risk of imatinib resistance emerging by reducing the number of tumour cells, since secondary mutations conferring drug resistance may arise by chance. However, further randomised trials are needed to investigate whether tumour bulk-reducing surgery is beneficial in selected patients being treated with imatinib for metastatic GIST.

Virtually all patients who receive imatinib have side effects, but severe adverse effects are infrequent and imatinib is generally considered well tolerated by the medical community. Yet, the patient`s perspective may differ from that of the physician`s perspective [14].

A significant proportion of patients with chronic myeloid leukaemia (CML) treated with imatinib and with long-term molecular remission remained in remission after imatinib discontinuation, suggesting that some CML patients might be cured with imatinib [15]. Importantly, of those who had molecular recurrence, almost all obtained a second remission after restarting of imatinib [15].

To our knowledge, no prospective study has investigated whether imatinib can be discontinued safely in patients who had surgical resection of all detectable metastatic lesions and who are in complete radiological remission after long-term imatinib treatment. If durable complete remissions could be achieved after imatinib discontinuation, the patients might be spared from imatinib-related side effects and costs [16]. We report the results of a prospective, multicentre phase II study that explored discontinuation of imatinib administration in patients with oligometastatic GIST.

## Patients and methods

### Study design and participants

The Scandinavian Sarcoma Group trial (SSG XXV; ClinicalTrials.gov identifier NTC02924714) is an open-label, single arm, prospective, multicentre phase II study, where eligible patients were assigned to discontinue imatinib. Patients were eligible if they were ≥18 of age; had immunohistochemically confirmed GIST and confirmed metastatic disease by radiology, histology or both in history; had >5.0 years of treatment with imatinib for metastatic disease excluding breaks in administration; had ≤3 detectable metastases in imaging during the course of the disease; had macroscopically complete resection (either R0 or R1 surgery) or radiofrequency ablation (RFA) of all metastases or had oligometastatic disease that disappeared completely on imatinib so that no remaining target lesions for surgery or RFA could be identified (including absence of residual cyst-like lesions) and had an Eastern Cooperative Oncology Group performance status ≤2. Patients were ineligible if they had metastases outside of the abdomen, GIST with a succinate dehydrogenase (*SDH*) mutation or other evidence for SDH deficiency, neurofibromatosis type 1, R2 resection of the primary tumour or metastasis or had progressive disease (PD) on imatinib or other systemic treatments for GIST before or after surgery/RFA of the metastases. The study protocol was approved by an institutional review board/ethics committee at the study sites. The study was conducted in accordance with the Declaration of Helsinki. All patients provided written informed consent before enrolment.

### Procedures

Patients assigned to discontinue imatinib were scheduled for follow-up visits consisting of physical examination, blood tests and imaging with computed tomography (CT) or MRI 2 months after imatinib discontinuation and then 3 monthly for the first year, 4 monthly for the second and the third years and 6 monthly for the fourth and the fifth years of follow-up or until PD. When disease progression occurred, radiological response to imatinib rechallenge was assessed using the Response Evaluation Criteria in Solid Tumours (RECIST) v1.1.

Patients were to complete a 3-level European Organisation for Research and Treatment of Cancer (EORTC) EQ-5D quality of life (QoL) questionnaire at screening and at every second scheduled follow-up visit. The EORTC QLC-EQ-5D questionnaires assess the following five health dimensions: mobility, self-care, usual activities, pain/discomfort and anxiety/depression. Each dimension has three levels: no problems, some problems and extreme problems. Patients were also asked to report the EuroQol-visual analogue scale (EQ VAS) from 0 (‘Worst imaginable health state’) to 100 (‘Best imaginable health state’).

### Survival

The primary endpoint was progression-free survival (PFS) 3 years after study entry. Secondary endpoints included OS and QoL compared to the baseline. PFS was defined as the time interval between the date of imatinib discontinuation and the date of first detection of GIST progression or death, whichever occurred first. Patients alive without progression were censored on the date of last follow-up. OS was calculated from the date of imatinib discontinuation until the date of death from any cause, censoring patients alive at the time of the last follow-up visit. Data collection was locked on September 1, 2022.

### Statistical analysis

The power of the study was estimated by examining PFS in the BRF-14 trial, where patients with metastatic GIST were randomised to continue or to discontinue imatinib [4, 5]. In the BRF-14 trial, discontinuation of imatinib after 1 year or 3 years of treatment led to rapid GIST recurrence/progression with 2-year PFS of 10 and 16%, respectively. Therefore, 3-year PFS was expected to be 15%, and an improvement to 35% was considered clinically significant. To find such an effect with 80% power using the 1-sided significance level of 0.05, 26 patients were needed for the study. To allow a drop-out rate of 15%, 31 patients were to be accrued (power 0.8, 1-sided alpha 0.05). Survival was estimated using the Kaplan-Meier method.

Change in the QoL was assessed by comparing the mean EQ VAS score between the baseline and the 5-month visit or the 11-month visit using the paired sample t-test Differences were considered statistically significant if the *p* values were <0.05.

## Results

### Patients

The trial closed prematurely due to slow accrual. Between January 5, 2017 and June 5, 2019, 13 patients were enrolled from three study sites (Oslo University Hospital, Norway, 10; Helsinki University Hospital, Finland, two; Karolinska University Hospital, Sweden, one). One patient was found ineligible because of too short time on imatinib treatment (<5 years) and was excluded from the analysis. The final study cohort thus consisted of 12 eligible patients, seven males and five females, with a median age of 67 (range, 50–85) at the time of study entry. All patients had metastases in the liver, the peritoneum or both. Patient baseline characteristics and GIST characteristics are provided in Table 1.

**Table 1:** Patient and GIST characteristics (N = 12).

Parameter	Number (%)
Sex (male:female)	7:5
ECOG performance status	
0	11 (92)
2	1 (8)
Primary tumour location	
Stomach	4 (33)
Small intestine	6 (50)
Colon/rectum	2 (8)
Longest primary tumour diameter, mm (range)	89 (22–240)
Tumour rupture	
No	11 (92)
Yes	1 (8)
Mutational type	
*KIT* exon 11	8 (67)
*KIT* exon 9	2 (17)
*PDGFRA* exon 12	1 (8)
No mutation detected in *KIT* or *PDGFRA*	1 (8)
Median mitotic count per 50 HPFs/5 mm^2^ (range)	14 (3–53)
Detection time of metastatic disease	
Synchronous with primary tumour	6 (50)
Metachronous	6 (50)
Metastatic sites	
Liver	8 (67)
Peritoneum	3 (25)
Liver and peritoneum	1 (8)
Number of metastases	
1	5 (42)
2	4 (33)
3	3 (25)
Median size of the largest metastatic lesion, mm (range)	34 (10–140)

ECOG: Eastern Cooperation Oncology Group; PDGFRA: Platelet derived growth factor alpha; HPF: high power field of the microscope; mm: millimeter.

### Prior treatments

All patients had undergone surgery at the time of primary diagnosis. Eight patients underwent surgical metastasectomy, two had RFA of a metastasis, one patient underwent both metastasectomy and RFA and one achieved complete radiological response with imatinib. One patient did not have a detectable *KIT/PDGFRA* mutation. Prior to the study, the patient had a radiological partial response to three liver metastases. He had resection of the metastases and the pathological report showed complete pathological response to the imatinib treatment. None of the patients had detectable GIST lesions at study entry. The median duration of imatinib treatment in the metastatic setting before the study entry was 8 years (range, 5–17). At enrolment, eight patients were taking imatinib 400 mg/day and four <400 mg/day.

### Survival outcomes

**Figure 1:**
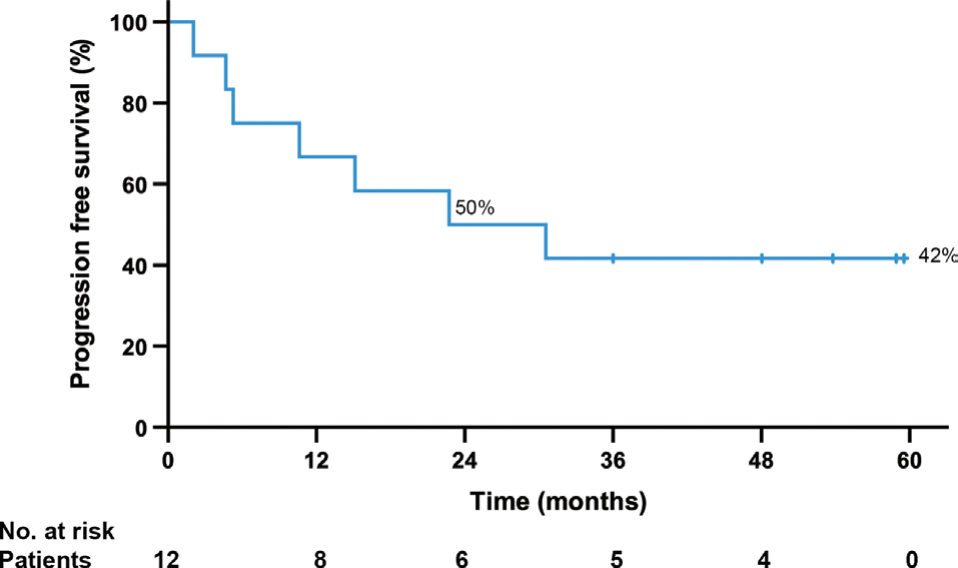
A Kaplan-Meier plot showing progression free-survival since the study entry. The patients without progression are indicated with a bar.

The median follow-up time was 55 months (range, 36 to 69 months) after study entry. All patients were alive at the time of the study analysis. Five (42%) of the 12 patients remained progression free (range, 36–60 months), and seven (58%) progressed with a median time to progression 10 months (range, 2–31 months). Median PFS was 23 months (range, 36–60 months), the estimated 3-year PFS 42% (Figure 1).

For the seven patients who progressed, five patients experienced intraperitoneal recurrence, and two patients progressed in the liver. Three of the four patients who had liver metastasis prior to the study entry recurred intraperitoneally and one in the liver. The two patients with prior intraperitoneal metastases: one progressed in that anatomical site and one in the liver. The patient with prior both liver and intraperitoneal metastases recurred in the liver.

**Figure 2:**
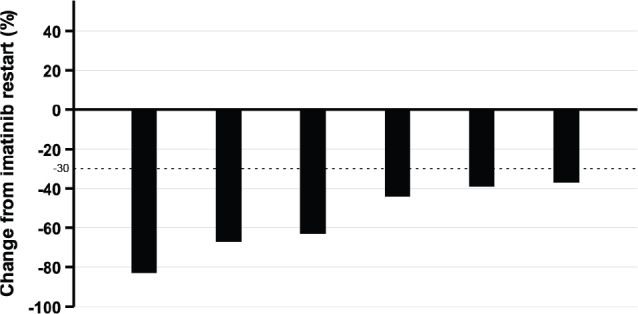
A waterfall plot showing best RECIST response after rechallenge with imatinib. Individual patients are represented with a vertical bar. Vertical axis: change in tumour size since the date of restarting imatinib.

Six of the seven patients who progressed restarted imatinib, and all six achieved a partial response (Figure 2). The only patient who did not restart imatinib at GIST progression was an 88-year-old woman with a single liver metastasis, and at the time of the data collection cut-off 16 months later, she had only minor progression of the metastasis without new lesions. Four of the six patients who restarted imatinib did not have disease progression during a median follow-up of 31 months (range, 19–39 months), whereas one patient with a *KIT* exon 9 mutation and another patient with a *KIT* exon 11 mutation had GIST progression 18 and 26 months after imatinib restart, respectively. The former patient was switched to second-line sunitinib, and the latter underwent stereotactic radiotherapy to a single progressive liver metastasis and was still on imatinib at the time of the data collection cut-off.

### QoL

**Figure 3:**
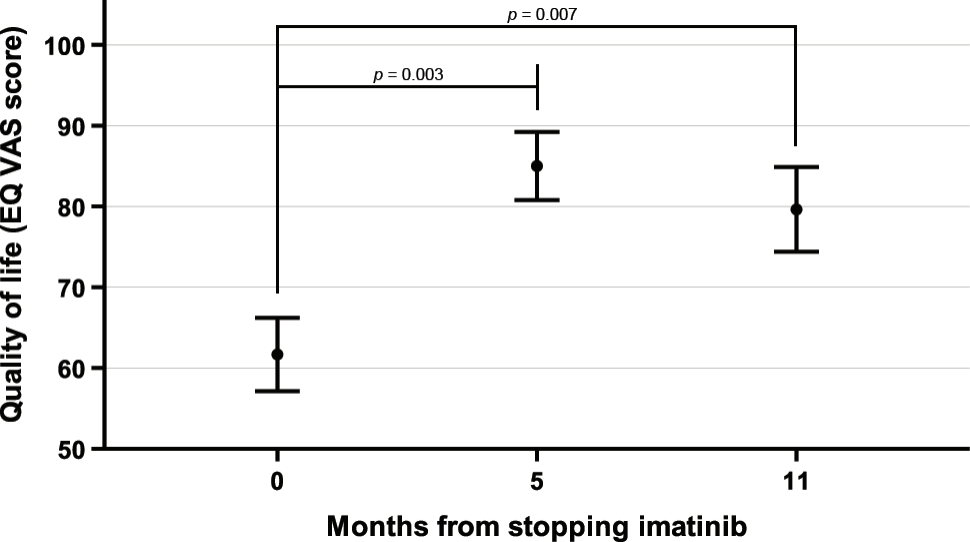
Patient-reported quality of life measured with the EQ visual analogue scale (VAS). The bullets represent the mean values and the whiskers one standard error of the mean.

All patients completed the 3-level QoL EQ-5D at baseline and 10 and nine patients at the 5- and 11-month follow-up visits, respectively. Few patients completed the QoL form at the subsequent visits prohibiting reliable analysis, and only few patients reported problems in the 5 QoL dimensions. The patients reported higher VAS scores indicating improved QoL 5 months after stopping imatinib compared with the baseline (mean = 85, standard error of mean [SEM] = 4.7 vs. mean = 65, SEM = 4.2; *p* = 0.003), and a difference to the baseline was maintained 11 months after stopping of imatinib (mean = 78, SEM 5.6 vs. mean = 63, SEM 4.7; *p* = 0.007; Figure 3). There was no statistically significant difference in the EQ VAS scores between the 5- and the 11-month visits.

## Discussion

A significant proportion of patients who continued to respond to imatinib and were rendered free from oligometastatic GIST with surgery or RFA remained free from detectable GIST for several years after stopping of imatinib administration. All patients who progressed after stopping and who restarted imatinib achieved a second radiological response. Judging from the EQ VAS scores, the QoL improved after stopping of imatinib, and none of the patients died during the follow-up.

Estimated 3-year PFS was 42%, which is higher than 3-year PFS of 16% observed in the BFR14 trial, where patients were randomised to continue or to discontinue first-line imatinib after planned time intervals on imatinib [4, 5]. Unlike in the BRF14 trial, we accrued only patients with oligometastatic disease who were in complete radiological remission on imatinib, and all patients except one had undergone metastasectomy or RFA of their metastases, the exception being a patient with a single liver metastasis who obtained complete remission on imatinib. The BFR14 trial patient population, in turn, was more unselected, local treatment of metastases was not required, and only a minority (0–5%) of the patients achieved complete response with imatinib, most responses being either partial responses or stable disease [4, 5]. A recent interesting retrospective study explored interruption of imatinib in 77 metastatic GIST patients who had been on imatinib for a median follow-up time of 72 months [17]. Here, the estimated 5-year PFS in this study was 26% and complete removal of residual disease was significantly associated with favourable PFS [17]. Dormant GIST cells are virtually always found in metastases or primary tumours excised from patients responding to imatinib [18]. Taken together, these observations suggest that discontinuation of imatinib as a treatment strategy may be best suited for patients with disease sensitive to imatinib and whose macroscopic metastases are removed surgically or treated successfully otherwise.

Oligometastatic GIST, defined as presence of ≤3 metastases when starting systemic treatment, is associated with favourable long-term survival compared to multimetastatic GIST [2]. In some cancer types, including sarcomas [7] and colorectal cancer [19], patients with oligometastatic disease are considered for metastasectomy with or without chemotherapy with curative intent, which leads to >30% 5-year disease-specific survival rates after complete resection. Oligometastatic disease has been regarded as an intermediate disease state between locoregional disease and widely metastatic disease; hence some patients are considered for curative treatment [20]. The present findings suggest a similar treatment strategy for selected patients with oligometastatic GIST.

While the median PFS of patients treated with first-line imatinib is around 2 years [21], the median time on imatinib before enrolment was 8 years in the present study. Thus, we included only patients with long-term benefit from imatinib, but it is unknown whether requiring long prior imatinib treatment was necessary. The SSGXVIII/AIO trial found 3 years of adjuvant imatinib to yield superior recurrence-free survival and OS rates compared with 1 year of imatinib [22], and the survival benefits persisted in study analyses based on 5- and 10-year median follow-up [23, 24], supporting a hypothesis that long administration of imatinib suppresses and perhaps sometimes eradicates micrometastatic disease.

All patients who restarted imatinib achieved a partial remission, which observation is compatible with observations in the BFR14 trial, where 92% of the patients responded to imatinib rechallenge [6]. In the Korean study mentioned above, the overall response rate was 88% with 100% disease control after reinstituting imatinib, and the median imatinib-refractory PFS was 112 months with an estimated 5-year PFS of 77% [17]. Moreover, in the BFR14 trial, the risk of imatinib resistance was comparable between the imatinib continuation and discontinuation groups, suggesting that imatinib discontinuation does not increase the risk for drug resistance mutations emerging early [25]. Imatinib discontinuation did not substantially influence OS in the BFR14 trial [4–6], and in the current study, all patients were alive after a median follow-up time of 55 months. Taken together, these findings suggests that imatinib discontinuation does not shorten OS in selected patients provided that the patients are followed up closely with longitudinal imaging.

Patients reported improved QoL after imatinib discontinuation. We interviewed nine of the patients included in the current study in a separate qualitative study [26] and found that as the adverse effects of imatinib disappeared the patients experienced positive changes in their daily lives and in their physical and mental health. This probably outweighed the anxiety caused by the fear of recurrence [26].

Continuous administration of first-line imatinib should be recommended for most patients with metastatic GIST. A randomised trial is needed to settle whether discontinuation of imatinib is a safe and effective treatment strategy for selected patients, but due to the relative rarity of responding and resectable oligometastatic disease and the long follow-up times needed, a randomised trial might not be carried out. Discontinuation of imatinib and close follow-up with imaging might be an option for selected patients who are in complete radiological remission after metastasectomy/RFA and imatinib administration and who experience adverse events from imatinib that compromise the QoL.

The study has some limitations, and due to the small patient numbers, the findings need to be viewed with caution. The main limitation is trial early closure due to slow accrual. Accrual was challenging in a relatively rare disease when stopping of imatinib in durable remission was met with some scepticism from patients and physicians, and the strict inclusion criteria excluded many patients from the study. All patients without progression were followed up at least for 3 years, but late recurrences might occur. In the absence of a contemporary control arm, QoL could not be compared with patients still on imatinib.

In conclusion, highly selected patients with oligometastatic GIST who have been rendered free of macroscopic disease with surgery, RFA or complete response on imatinib administration may survive with good QoL without detectable GIST for several years after imatinib discontinuation. While these results encourage further study on imatinib discontinuation, continuous imatinib administration remains the standard of care for first-line patients with imatinib-sensitive advanced GIST.

## Author contributions

IH: Conceptualisation, Writing – Original Draft, Formal analysis, Resources, Visualisation, Project administration.

KB: Conceptualisation, Formal analysis, Resources, Writing – Review and Editing.

AMW: Resources, Writing – Review and Editing.

BR: Conceptualidation, Resources, Writing – Review and Editing.

AP: Resources, Writing – Review and Editing.

HJ: Conceptualisation, Resources, Writing – Review and Editing, Supervision.

ØB: Conceptualisation, Writing – Original Draft, Formal analysis, Resources, Visualisation, Supervision, Project administration.

## Disclosures

KB has reported honoraria for expert testimony and advisory boards from Bayer, honoraria for advisory boards from GSK, invited speaker fees and institutional research support from Eli Lilly, invited speaker fees from Novartis and institutional research support from Merck; HJ has reported a leadership role in Orion Pharma, Neutron Therapeutics and Sartar Therapeutics, consulting/advisory role in Orion Pharma and Neutron Therapeutics, honoraria for scientific meetings from Deciphera Pharmaceuticals and has stock ownership in Orion Pharma and Sartar Therapeutics; IH, AMW, BR, AP and OSB have declared no conflicts of interest.

## Data availability statement

The raw data supporting the conclusions of this article can be made available by the authors, without undue reservations.

## Ethics declarations

The study protocol was approved by an institutional review board/ethics committee at the study sites. The study was conducted in accordance with the Declaration of Helsinki. All patients provided written informed consent before enrolment. Trial registry identifiers/approval numbers in ClinicalTrials.gov was NTC02924714.
